# Interfacial Recognition of Acetylcholine by an Amphiphilic *p*-Sulfonatocalix[8]arene Derivative Incorporated into Dimyristoyl Phosphatidylcholine Vesicles

**DOI:** 10.3390/s8106777

**Published:** 2008-10-29

**Authors:** Takashi Jin, Fumihiko Fujii, Yasuhiro Ooi

**Affiliations:** 1 Nano-bio Materials Laboratory, Immunology Frontier Research Center, Osaka University, Suita, Osaka, 565-0871, Japan; E-mail: fujii@fbs.osaka-u.ac.jp; 2 Graduate School of Frontier Biosciences, High Performance Bioimaging Facility, Suita, Osaka University, Osaka 565-0871, Japan.; 3 Division of Pathogenesis and Control of Oral Disease, Graduate School of Dentistry, Osaka University, Suita, Osaka, 565-0781, Japan; E-mail: ymgtooi@fbs.osaka-u.ac.jp

**Keywords:** Interfacial recognition, acetylcholine, sulfonatocalixarene, lipid bilayer, vesicle, fluorescence correlation spectroscopy

## Abstract

Dodecyl ether derivatives **1-3** of *p*-sulfonatocalix[n]arene were incorporated into dimyristoyl phosphatidylcholine (DMPC) vesicles, and their binding abilities for acetylcholine (ACh) were examined by using steady-state fluorescence/fluorescence anisotropy and fluorescence correlation spectroscopy (FCS). For the detection of ACh binding to the DMPC vesicles containing 5 mol % of **1-3**, competitive fluorophore displacement experiments were performed, where rhodamine 6G (Rh6G) was used as a fluorescent guest. The addition of Rh6G to the DMPC vesicles containing **3** resulted in a decrease in the fluorescence intensity of Rh6G with an increase of its fluorescence anisotropy, indicating that Rh6G binds to the DMPC-**3** vesicles. In the case of DMPC-**1** and DMPC-**2** vesicles, significant changes in the fluorescence spectra of Rh6G were not observed. When ACh was added to the DMPC-**3** vesicles in the presence of Rh6G ([**3**]/[Rh6G]=100), the fluorescence intensity of Rh6G increased with a decrease in its fluorescence anisotropy. From the analysis of fluorescence titration data, the association constants were determined to be 7.1×10^5^ M^-1^ for Rh6G-**3** complex and 1.1×10^2^ M^-1^ for ACh-**3** complex at the DMPC-**3** vesicles. To get a direct evidence for the binding of Rh6G and its displacement by ACh at the DMPC-**3** vesicles, diffusion times of the Rh6G were measured by using FCS. Binding selectivity of the DMPC-**3** vesicles for ACh, choline, GABA, l-aspartic acid,l-glutamic acid, l-arginine, l-lysine, l-histamine and ammonium chloride was also evaluated using FCS.

## 1. Introduction

Ligand-receptor interaction in biological membranes plays important roles in the initiation of signal transduction [1]. For instance, signal transduction in nerve systems is mediated by neurotransmitters that bind to the receptor at postsynaptic membranes. In biological systems, most of the receptors work at the water-membrane interface, where the receptors are bound at lipid bilayer membranes. Over the past two decades, much effort has gone into the synthesis of artificial neurotransmitter receptors using host molecules such as cryptophanes [2, 3], calixarenes [4-10] and resorcinarenes [11-15]. However, the synthesis of artificial neurotransmitter receptors that work at lipid bilayer membrane systems is still challenging. In this paper, we report an artificial neurotransmitter receptor based on an amphiphilic derivative of *p*-sulfonatocalix[8]arene that recognizes acetylcholine (ACh) at a water-membrane interface of dimyristoyl phosphatidylcholine (DMPC) vesicles.

So far, it has been shown that water-soluble *p*-sulfonatocalix[n]arenes have binding abilities for quaternary ammonium-based cations [8, 16-19], and they are able to recognize ACh in aqueous solution [4-10, 20]. Lehn *et al.* [4] reported that *p*-sulfonatocalix[4]arene and *p*-sulfonatocalix[6]arene show high affinities for choline and ACh, where the association constants are comparable to those of the biological recognition sites. Koh *et al.* [5] reported an artificial ACh detection system using a complex of a fluorescent guest and *p*-sulfonatocalix[6]arene. Zhang *et al.* [6] and our group [7] showed that the binding affinities of ACh in aqueous solution increases in the order of *p*-sulfonatocalix[4]arene < *p*-sulfonato-calix[6]arene < *p*-sulfonatocalix[8]arene based on competitive fluorophore displacement experiments.

In this work, we have studied binding properties of amphiphilic *p*-sulfonatocalix[n]arene in a DMPC lipid bilayer membrane system. A lipid bilayer membrane consists of amphiphilic lipids, where the lipid bilayer is very thin and a molecular-sized membrane (< 1 nm). To incorporate *p*-sulfonatocalix[n]arenes to the lipid bilayer membranes, the calix[n]arenes should be modified to have amphiphilic nature. Since the *p*-sulfonatocalix[n]arenes have phenolic hydroxyl groups, the derivation with alkyl chains to amphiphilic compounds is easily performed. We synthesized dodecyl ethers of *p*-sulfonatocalix[n]arenes **1-3** that are able to be incorporated into DMPC vesicles. Since the DMPC has a similar molecular length with **1-3**, it was expected that the mixture of **1-3** and DMPC would form stable lipid bilayer membranes, and the ACh binding moiety [4-7] of **1-3** would locate at the surface of the vesicles. The binding abilities of the DMPC vesicles containing **1-3** for ACh are demonstrated by the measurements of steady state fluorescence spectra, fluorescence anisotropy and fluorescence correlation spectroscopy (FCS).

## 2. Results and Discussion

### 2.1 The binding ability and the stoichiometry of binding of parent p-sulfonatocalix[n]arenes for Rh6G and ACh in aqueous solution

**Scheme 1.**
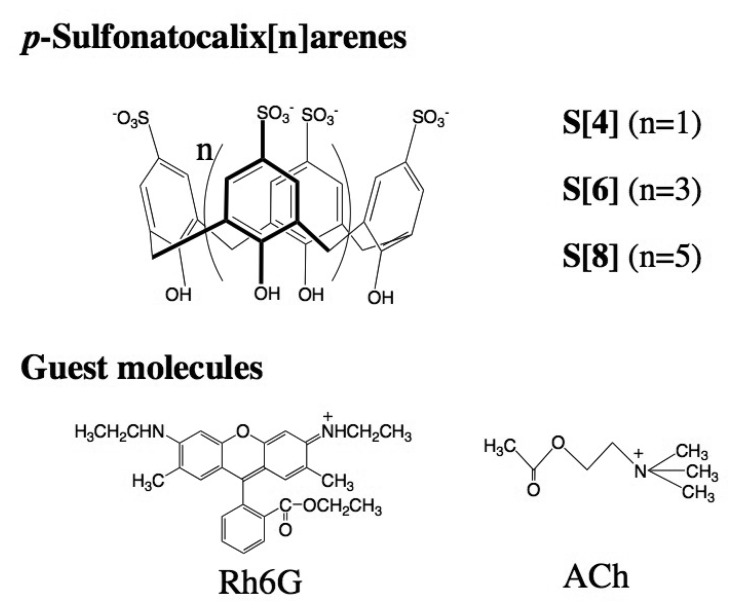
Molecular structure of *p*-sulfonatocalix[n]arenes and guest molecules, rhodamine 6G (Rh6G) and acetylcholine (ACh).

Previously we have reported that *p*-sulfonatocalix[n]arenes form complexes with ACh in aqueous solution on the basis of ^1^H-NMR and fluorescence measurements using dancylcholine as a fluorescent ACh analogue [7]. We have shown that among the *p*-sulfonatocalix[n]arenes, *p*-sulfonatocalix[8]arene has a highest binding ability for ACh with a high selectivity. In this study we first examined the complexing properties of *p*-sulfonatocalix[n]arenes (S[4], S[6], and S[8]) for Rh6G and ACh in aqueous solution (Scheme 1), because the dodecyl ether derivatives of S[n] showed poor water-solubilities and they did not solubilize to be a homogenous aqueous solution.

**Figure 1.**
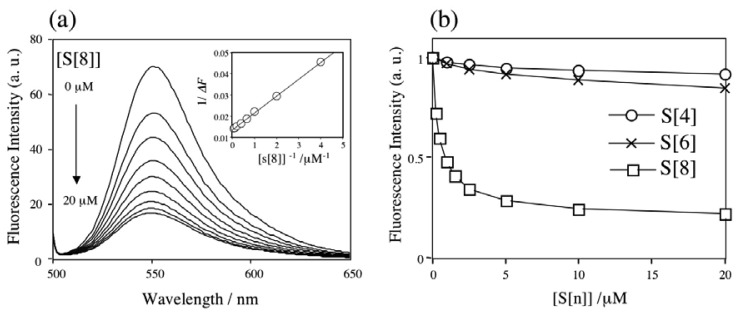
Fluorescence change of Rh6G by complexion with S[n] in 10 mM phosphate buffer (pH = 6.86). (a) Changes in the fluorescence spectra of Rh6G upon adding S[8]. Inset shows a plot of *1/ΔF* and *1/[S[8]]*. (b) Dependence of the fluorescence intensity of Rh6G on the concentration of S[n].

Figure 1a shows changes in the fluorescence spectra of Rh6G (20 nM in 10 mM phosphate buffer) when S[8] was added to the Rh6G solutions. The fluorescence of Rh6G was quenched by S[8], indicating the formation of Rh6G-S[8] complexes. Similar fluorescence quenching was reported by Zhang *et al.* in the case of (rhodamine B)-S[8] complex [6]. Assuming the formation of a 1:1 stoichiometry for the Rh6G-S[8] complex, its association constant *K_Rh6G_* could be determined by analysis of the fluorescence intensity change (*ΔF*) [7]:

(1) $$\frac{1}{\Delta F}=\frac{1}{c}+\frac{1}{c\cdot K_{\text{Rh}6G}\cdot [S[n]]}$$

where *c* is a constant. A plot of *1*/*ΔF* vs. *1*/*[S[8]]* showed a linear relationship, suggesting that Rh6G binds S[8] with a 1:1 stoichiometry (inset in Figure 1a). For S[4] and S[6], we also observed similar linear relationships for the plot of *1*/*ΔF* and *1*/*[S[n]]* (data not shown) and confirmed the formation a 1:1 complex of Rh6G with S[4] and S[6]. Figure 1b shows changes in the fluorescence intensity of Rh6G upon adding S[n]. From the Equation (1), the association constants were determined to be 1.9 × 10^3^, 4.4×10^3^, and 1.9×10^6^ M^-1^ for the Rh6G complexes with S[4], S[6], and S[8], respectively. The values of the association constants indicate that the binding of Rh6G-S[8] is stronger than that of Rh6G-S[4] and Rh6G-S[6] by a factor of 1000 and 431, depending on the size of the cavity of S[n].

**Figure 2.**
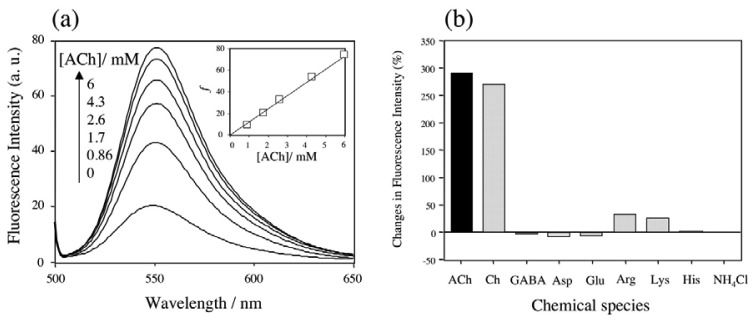
Fluorescence change of Rh6G (20 nM) by addition of ACh in the presence of 20 μM of S[8] in 10 mM phosphate buffer (pH = 6.86). (a) Changes in the fluorescence spectra of Rh6G upon adding ACh. Inset shows a plot of *f* and 1/[ACh]. (b) Fluorescence intensity change (%) of Rh6G by addition of ACh and other chemicals (6 mM): Ch; choline, GABA; 4-aminobutylic acid, Asp; l-asparatic acid, Glu; l-glutamic acid, Agr; l-arginine, Lys; l-lysine, His; l-histamine, and NH_4_Cl; ammonium chloride.

The binding ability of ACh by S[8] was confirmed by a competitive fluorophore displacement experiment. Figure 2a shows changes in the fluorescence spectra of Rh6G by addition of ACh, where the ratio of S[8]/Rh6G is 1000. The fluorescence intensity of R6G increases with increasing the concentration of [ACh], indicating that ACh competitively binds to the Rh6G complex, and Rh6G is replaced with ACh. Two association constants (*K_Rh6G_* and *K_ACh_*) can be rationalized by the following equation under the condition of ACh in excess [7]:

(2) $$\begin{array}{cc} \frac{\alpha \cdot ([S[8]]-(1-\alpha )[\text{Rh}6G])\cdot K_{\text{Rh}6G}}{1-\alpha }=1+{\left[\text{ACh}\right]}^{n}\cdot K_{\text{ACh}}, & \;\alpha =(F-F_{o})(F_{\infty }-F_{o}) \end{array}$$

where *F_o_*, *F*, and *F_∞_* are the fluorescence intensities for the Rh6G emission measured before, during, and after the titration with ACh to a saturation concentration, and *n* is a number of binding molecules of ACh to S[8].

The inset in Figure 2a shows a plot of *f* (=*α([S[8]]*-*(1*-*α)[Rh6G])K_Rh6G_*/*(1*-*α))* and *[ACh]*. A linear relationship indicates that ACh forms a 1:1 complex with S[8]. The association constant of *K_ACh_* is determined to be 1.3 × 10^4^ M^-1^, showing that the binding affinity of ACh for S[8] is 146 times lower than that of Rh6G for S[8]. The binding selectivity of S[8] could be evaluated from the fluorescence intensity changes caused by the competitive fluorophore displacement. Figure 2b shows the changes in the fluorescence intensity of Rh6G by adding ACh and other chemicals. This result suggests that S[8] selectively binds to ACh and choline with their quaternary ammonium moieties. The increase in the fluorescence intensity for l-arginine and l-lysine indicates that S[8] can also bind their primary ammonium cations.

### 2.2 Characterization of DMPC vesicles containing dodecyl ether derivatives of p-sulfonato-calix[n]arenes **1-3**

Since parent *p*-sulfonatocalix[n]arenes (S[n]) are highly hydrophilic compounds, they cannot be incorporated into lipid bilayer membranes. Lipid bilayer membranes consist of amphiphilic lipids, and they are very thin and molecular-size membranes [21]. To incorporate *p*-sulfonatocalix[n]arenes to the lipid bilayer membranes, we modified hydroxyl groups of the *p*-sulfonatocalix[n]arenes with dodecyl alkyl chains to be amphiphilic compounds. The resulting derivatives **1-3** showed poor solubilities in water, while they showed good solubilities to organic solvents such as dimethyl sulfoxide.

**Scheme 2.**
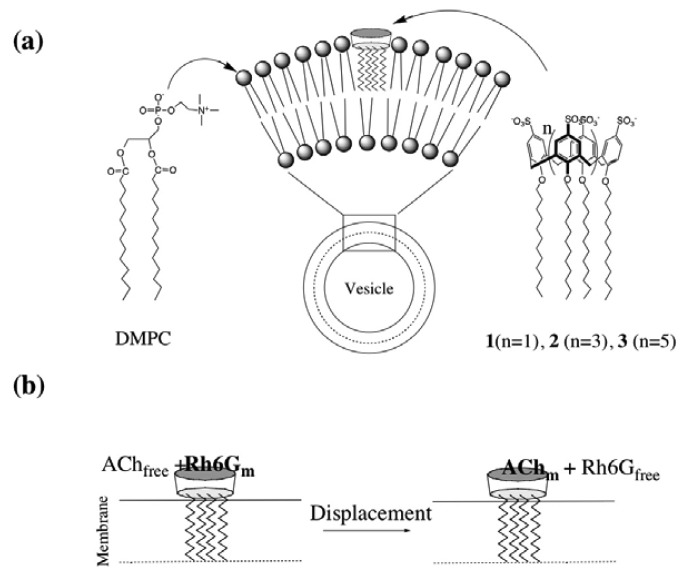
(a) Schematic representation of a DMPC vesicle containing *p*-sulfonatocalix-[n]arene derivatives **1-3**. (b) Displacement of Rh6G bound at DMPC-(**1-3**) vesicles by ACh.

As lipids for the preparation of bilayer membrane vesicles, we chose dimyristoyl phosphatidylcholine (DMPC) that has a hydrophilic head group consisting of a choline-phosphate moiety and two hydrophobic alkyl chains. Since the DMPC has a similar molecular length as **1-3** (Scheme 2a), it was expected that the mixture of **1-3** and DMPC would form stable lipid bilayer membranes, and the *p*-sulfonatocalixarene moieties of **1-3** would be positioned on the membrane surface. For the conformation of **1-3**, we confirmed that they form a cone conformation at room temperature on the basis of the ^1^H-NMR spectra of their bridging methylene protons in dimethyl sulfoxide (see Experimental section). The DMPC vesicles containing **1-3** were prepared by sonication of the mixture of DMPC and 5 mol % of **1-3** in phosphate buffer at 50 °C. The size of the DMPC vesicles containing **1-3** was measured using a dynamic light scattering (ca. 30 nm in diameter). To confirm the incorporation of **1-3** to the DMPC vesicles, we measured zeta potentials. Table 1 shows the values of zeta potentials for DMPC vesicles and DMPC vesicles containing 5 mol % of **1-3** in 10 mM phosphate buffer (pH = 6.86). The zeta potential for the DMPC vesicles shows the value of ca. + 2 mV, whereas negative values of the zeta potentials from -7.1 to -10.1 mV are obtained for DMPC vesicles containing **1-3**. This finding indicates that *p*-sulfonatocalix[n]arene derivatives **1-3** are incorporated into the DMPC vesicles and the sulfonyl groups of **1-3** are located at the surface of vesicles (Scheme 2a).

### 2.3 Fluorescence spectra and fluorescence anisotropy measurements for the binding of Rh6G and ACh to DMPC vesicles containing **1-3**

**Figure 3.**
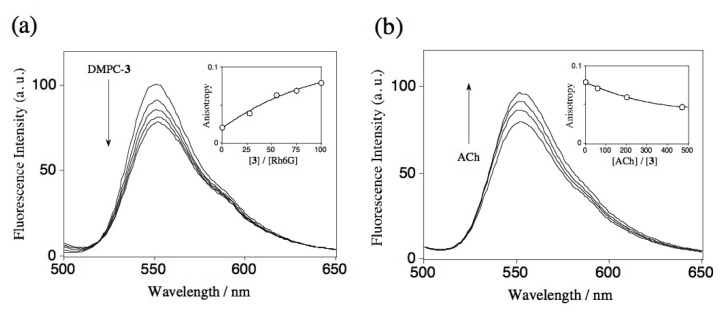
Fluorescence spectra of Rh6G (20 nM) in 10 mM phosphate buffer (pH = 6.86). (a) Addition of DMPC vesicles (5 mg/20 mL) containing 5 mol % of **3**. (b) Addition of ACh to the mixture of Rh6G and DMPC-**3** vesicles ([**3**]/ [Rh6G] = 100). Inset shows changes in the fluorescence anisotropy of Rh6G.

To examine the binding abilities of ACh by DMPC vesicles containing **1-3**, competitive fluorophore displacement experiments using Rh6G were performed (Scheme 2b). We measured a steady-state fluorescence of Rh6G in the presence of DMPC vesicles containing **1-3** to check their binding abilities for Rh6G. Figure 3a shows the fluorescence spectra of Rh6G (20 nM) upon adding DMPC-**3** vesicles. The fluorescence intensity of Rh6G is quenched with increasing the concentration of DMPC-**3** vesicles. In the case of the DMPC vesicles containing **1** and **2**, the addition of these vesicles causes only a slight change in the fluorescence spectra under the same experimental condition (data not shown). To confirm the binding of Rh6G to the DMPC-**3** vesicle, we measured fluorescence anisotropy [22] of Rh6G. The inset in Figure 3a shows the dependence of fluorescence anisotropy on the ratios of [**3**]/[Rh6G]. Upon adding DMPC-**3** vesicles to the Rh6G solution, the fluorescence anisotropy increases, indicating that Rh6G binds to the DMPC-**3** vesicle membrane. Figure 3b shows the fluorescence spectra of Rh6G when ACh was added to the mixture of Rh6G and DMPC-**3** vesicles ([**3**]/[Rh6G]=100). In this case, the fluorescence intensity of Rh6G increases and its fluorescence anisotropy decreases with increasing the concentration of ACh. This result suggests that Rh6G molecules bound at the DMPC-**3** vesicle are replaced with ACh as shown in Scheme 2b. Based on the equations of (1) and (2), association constants for Rh6G-**3** and ACh-**3** complexes at DMPC membranes are determined to be 7.1×10^5^ M^-1^ and 1.1×10^2^ M^-1^, respectively. In the DMPC-**3** system, fluorescence change in response to the binding of Rh6G is small in compared with the case of the homogeneous solution system used S[8] (Figure 1a). Even in the presence of excess amounts of DMPC-**3** vesicles, the fluorescence of Rh6G was not completely quenched. This finding may suggest that a Rh6G molecule is not deeply included in the cavity of **3** in the DMPC-**3** vesicle system.

It should be noted that the association constant of ACh-**3** complexes at DMPC membrane is 118 times lower than that of ACh-S[8] complexes in aqueous solution. Large reduction of the association constant in vesicle membranes has been reported for the case of the K^+^ complexes with valinomycin and nigericin [23]. The reduction of the association constant of ACh-**3** complexes may be attributed to the strong interaction between quaternary ammonium moieties of ACh and the phosphate groups of DMPC molecules at a water-membrane interface.

### 2.4 Fluorescence correlation spectroscopy for the binding of Rh6G and ACh to DMPC vesicles containing **1-3**

To get a direct evidence for the binding of ACh to DMPC-**3** vesicles, we used fluorescence correlation spectroscopy (FCS) [24]. FCS is a powerful method to monitor changes in the translational diffusion rates of fluorescent molecules in solution. Assuming that fluctuation of the fluorescence intensity of Rh6G arises only from translational diffusion, time-dependent part of the autocorrelation function *G(τ)* [24, 25] for multiple fluorescent species is given by:

(3) $$G(\tau )=1+\frac{1}{N}\cdot \sum_{i=1}^{M}f_{i}{\left(1+\frac{\tau }{\tau_{D_{i}}}\right)}^{-1}{\left(1+s^{-2}\left(\frac{\tau }{\tau_{D_{i}}}\right)\right)}^{-\frac{1}{2}}$$

where *N* and *f_i_* are the total number and fraction of fluorescent species in the confocal volume, respectively; *τ_Di_* is the diffusion time of the fluorescent species and *s* is a structure parameter describing the ratio of length vs. diameter of the confocal volume.

**Figure 4.**
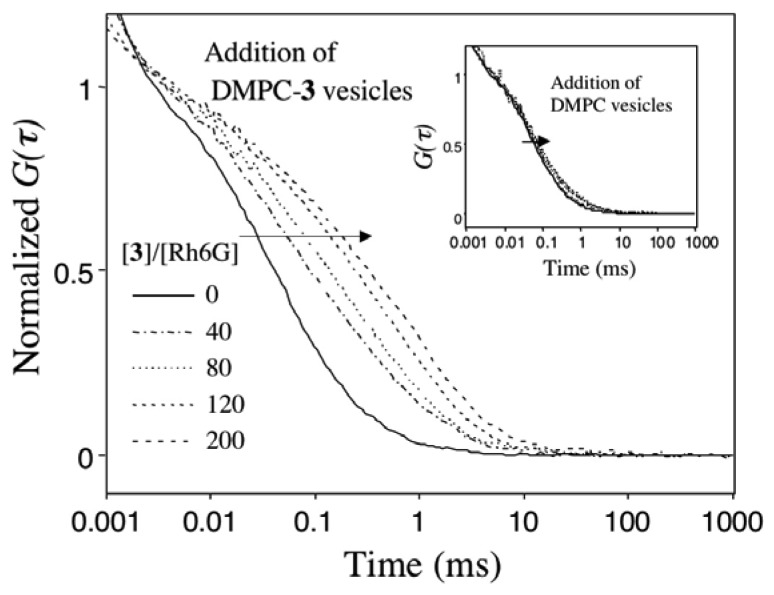
Fluorescence autocorrelation curves *G(τ)* of Rh6G (20 nM) in 10 mM phosphate buffer (pH = 6.86) upon adding DMPC-**3** vesicles. Inset shows a control experiment where DMPC vesicles (5 mg/20 mL) were added to the Rh6G solution

Figure 4 shows the *G(τ)* curves of Rh6G (20 nM) upon adding DMPC vesicles containing 5 mol % of **3**. The *G(τ)* curve of Rh6G in the absence of the DMPC-**3** vesicles is fitted to a single-component diffusion model, and the diffusion time of free Rh6G is calculated to be 0.04 ms. When the DMPC-**3** vesicles are added to the aqueous solution of Rh6G, the *G(τ)* curve of Rh6G shifts to the right side with longer diffusion times.

This shift of the *G(τ)* curve indicates that Rh6G binds to the DMPC-**3** vesicle membranes. The *G(τ)* curves of Rh6G in the presence of DMPC-**3** vesicles can be fitted to a two-component diffusion model where the diffusion times of free and membrane-bound Rh6G are 0.04 and 0.9 ms, respectively. In the control experiment, addition of DMPC vesicles that do not contain **1**-**3** caused only a slight change in the *G(τ)* curve of Rh6G (inset).

**Figure 5.**
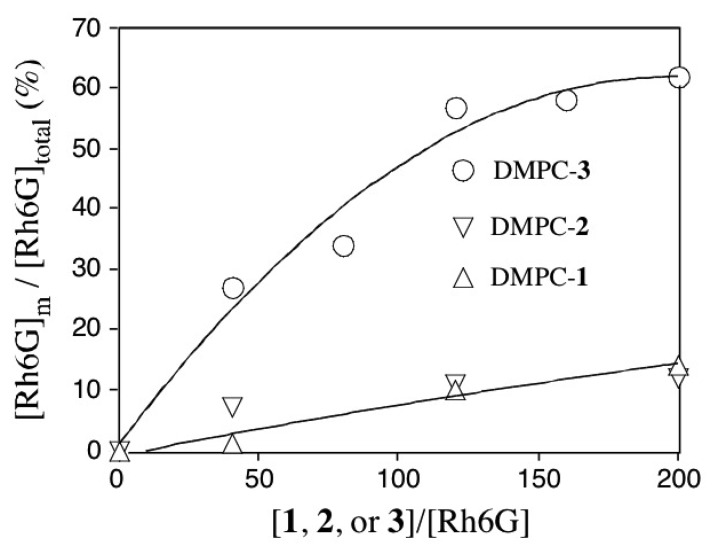
Fraction of [Rh6G]_m_ in the presence of DMPC vesicles containing 5 mol % of **1-3**. [Rh6G]_total_ = 20 nM.

The fractions of free and membrane-bound Rh6G are calculated by the fit of *G(τ)* curves to Equation (3). Figure 5 shows the fraction of membrane-bound Rh6G ([Rh6G]_m_) in the presence of DMPC vesicles containing **1-3**. When DMPC-**3** vesicles are added to the aqueous solution of Rh6G, the fraction of [Rh6G]_m_ significantly increases. In the presence of excess amounts of DMPC-**3** vesicles ([**3**]/[Rh6G]=200), ca 60 % of Rh6G molecules is bound to the DMPC-**3** membranes. When DMPC-**1** or DMPC-**2** vesicles are added, ca. 10 % of Rh6G molecules bind to the vesicles. In this case, however, the fraction of membrane-bound Rh6G is almost the same as those in the control experiment using DMPC vesicles.

**Figure 6.**
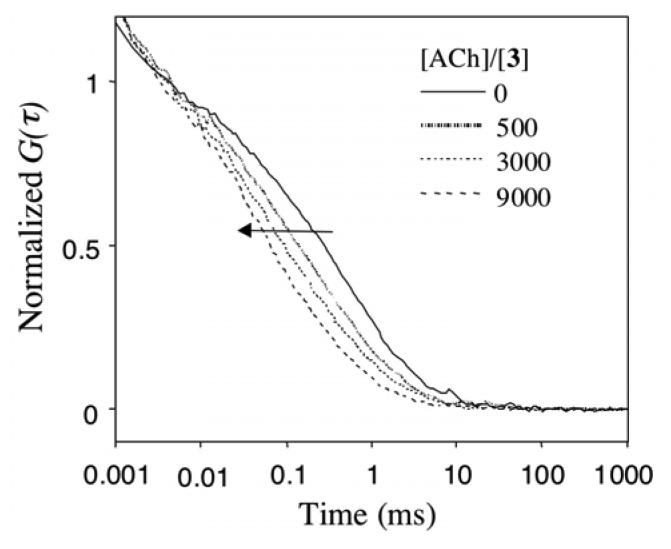
The effects of ACh on the fluorescence correlation curve *G(τ)* of Rh6G in the presence of DMPC-**3** vesicles ([**3**]/[Rh6G] =80, [Rh6G]=20 nM).

Figure 6 shows the effects of ACh on the *G(τ)* curve of Rh6G in a DMPC-**3** vesicle dispersion. The *G(τ)* curve in the absence of ACh is fitted to a two component model, where 55 % of Rh6G is bound to the DMPC-**3** vesicles and 45 % of Rh6G is free in solution. Upon addition of ACh, the *G(τ)* curve shifts to the left side with a decrease in diffusion times of Rh6G, indicating that Rh6G molecules bound at the DMPC-**3** vesicle are replaced with ACh, and the fraction of free Rh6G is increased. In the presence of 9000 amounts of ACh over **3**, ca. 50 % of membrane-bound Rh6G is replaced with ACh.

### 2.5 The binding selectivity of DMPC-**3** vesicle

**Figure 7.**
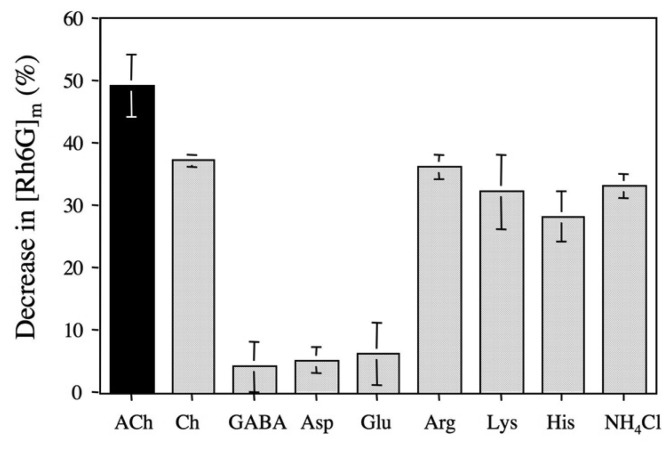
Decrease in [Rh6G]_m_ (%) by addition of ACh and other chemicals (9000-fold amounts of **3**) in the presence of DMPC-**3** vesicles ([**3**]/[Rh6G] = 80, [Rh6G] = 20 nM): Ch; choline, GABA; 4-aminobutylic acid, Asp; l-asparatic acid, Glu; l-glutamic acid, Agr; l-arginine, Lys; l-lysine, His; l-histidine, and NH_4_Cl; ammonium chloride.

The binding selectivity of DMPC-**3** vesicle was evaluated from by the changes of membrane-bound Rh6G ([Rh6G]_m_) upon adding ACh and other chemicals. Figure 7 shows decrease in the [Rh6G]_m_ of DMPC-**3** vesicle solutions as a result of a competitive fluorophore displacement. The additions of ACh and choline cause significant decreases in the fraction of [Rh6G]_m_, while the additions of anionic and neutral neurotransmitters such as l-glutamic acid, l-aspartic acid and GABA slightly affect the [Rh6G]_m_. This dependency of the binding selectivity is similar to that of the parent *p*-sulfonatocalix[8]arene in aqueous solution (Figure 2b). However, the addition of basic amino acids (l-arginine, l-lysine, and l-histidine) and ammonium cation also significantly decreases the fraction of [Rh6G]_m_. The binding affinity among the basic amino acids decreases in the order of l-arginine > l-lysine > l-histidine. Interestingly, this order is consistent with that of the decrease in the basicity of the amino acids: pK values of *l*-arginine, l-lysine, and l-histidine are 12, 10.2 and 6.5, respectively [26]. This finding suggests that the membrane potential of a negative value (ca. -7 mV) of DMPC-**3** vesicle may induce the interaction between basic amino acids and the vesicle surface. In the case of ACh, hydrophobic interaction as well as electrostatic interaction between the quaternary ammonium moiety of ACh and a π-basic cavity of **3** should play an important role in the binding by DMPC-**3** vesicle [4, 7, 17].

## 3. Experimental Section

### 3.1. Materials and Methods

*p*-Sulfonatocalix[4]arene and *p*-sulfonatocalix[6]arene were purchased from Tokyo Organic Chemicals (Japan). *p*-Sulfonatocalix[8]arene was purchased from Dojin Chemicals (Japan). Dodecyl bromide was purchased from Wako Chemicals (Japan). DMPC (1,2-dimyristoyl-rac-glycero-3-phospho-choline) was purchased from Sigma-Aldrich. Other chemicals used were analytical grade. ^1^H NMR spectra were measured on a Bruker AVANCE 500 WB spectrometer. ESI-MS spectra were measured on a JEOL JMS-700TZ spectrometer.

### 3.2. Synthesis

The synthesis of **1-3** was carried out according to the literature method [27]. A typical procedure for the synthesis of **3** is as follows: the octasodium salt of *p*-sulfonatocalix[8]arene (1 g, 0.6 mmol) was mixed with NaOH (1 g, 25 mmol) in water (5 mL) and dodecyl bromide (5 g, 20 mmol) in dimethyl sulfoxide (20 mL), and the reaction was heated at 60 °C for 24 h. After cooling, the solution was diluted with methanol to precipitate the product. The precipitate was recovered by filtration and dissolved in water (5 mL). After an insoluble material was removed by filtration, the product was precipitate from the filtrate by diluting with ethanol. This operation was repeated three times. Compound **1**: ^1^H-NMR (500 MHz, Me_2_SO-d_6_, 25 °C): *δ* 0.83 (CH_3_, t, 12H), 1.21 (Me(CH_2_)_8_, s, 64H) , 1.35 (O-C-CH_2_-CH_2_-*CH_2_*, s, 8H), 1.91 (O-C-CH_2_-*CH_2_*, s, 8H), 3.20 (ring CH_2_, d, *J* =13.0 Hz, 4H), 3.86 (O-C-*CH_2_*, t, 8H), 4.34 (ring CH_2_, d, *J* =13.0 Hz, 4H), 7.10 (Ar H, s, 8H); ESI-MS: calcd. M=1504 for C_76_H_116_O_16_S_4_Na_4_; found, 729.34 [M-2Na]^2-^; Compound **2**: ^1^H-NMR (500 MHz, Me_2_SO-d_6_, 45 °C): *δ* 0.85 (CH_3_, t, 12H), 1.26 (Me(CH_2_)_7_, s, 84H) , 1.42 (O-C-CH_2_-CH_2_-CH_2_-*CH_2_*, s, 12H), 1.51 (O-C-CH_2_-CH_2_-*CH_2_*, s, 12H), 1.80 (O-C-CH_2_-*CH_2_*, s, 12H), 3.38 (ring CH_2_, d, *J* =14.7 Hz Hz, 6H), 3.65 (O-C-*CH_2_*, s, 8H), 4.54 (ring CH_2_, d, *J* =14.7 Hz, 6H), 7.37 (Ar H, s, 8H); ESI-MS: calcd. M=2257 for C_114_H_174_O_24_S_6_Na_6_ (M=2257); found, 1105.98 [M-2Na]^2-^; Compound **3**: ^1^H-NMR (500 MHz, Me_2_SO-d_6_, 45 °C): *δ* 0.84 (CH_3_, t, 24H), 1.24 (Me(CH_2_)_7_, s, 128H) , 1.43 (O-C-CH_2_-CH_2_-*CH_2_*, s, 16H), 1.61 (O-C-CH_2_-*CH_2_*, s, 16H), 3.62 (ring CH_2_, d, *J* =16.3 Hz, 8H), 3.54 (O-C-*CH_2_*, s, 16H), 4.31 (ring CH_2_, d, *J* =16.3 Hz, 8H), 7.26 (Ar H, s, 16H); ESI-MS: calcd. M=2257 for C_152_H_232_O_32_S_8_Na_8_; found, 1105.98 [M-2Na]^2-^.

### 3.3. Preparation of DMPV vesicles containing **1-3**

The mixture of 5 mg of DMPC and 5 mol % of **1**, **2**, or **3** was dissolved in tetrahydrofuran (1 mL) and the solvent was evaporated under reduced pressure. Then, 10 mM phosphate buffer (pH= 6.86, 20 mL) was added and the dispersion was sonicated for 20 min at 50 °C (Branson, sonifier-150). After the vesicle dispersion became to be clear, the solution was passed through 0.2 μM membrane filter and dialyzed by the phosphate buffer.

### 3.4. Fluorescence spectra and fluorescence anisotropy measurements

Fluorescence spectra were measured on a Shimadzu RF-5300PC spectrofluorophotometer using a quarts cuvettz (1 cm × 1 cm × 4.5 cm), where excitation wavelength was set at 480 nm. Fluorescence anisotropy *r* of Rh6G was measured at 550 nm at 25 °C: *r*=*(I_‖_*-*I_⊥_)*/*(I_‖_*+*2I_⊥_)*, where *I_‖_* and *I_⊥_* are the parallel and perpendicular polarized intensity of fluorescence in respect to the polarized excitation [21].

### 3.5. Vesicle size and zeta potential measurements

The vesicle size and zeta potential were measured by using a dynamic light scattering apparatus (Malvern, Nano-ZS). A He-Ne laser (633 nm) was used as a light source. The diameter of vesicles was determined to be 27 ± 4, 27 ± 7, and 29 ± 7 nm for DMPC-**1**, DMPC-**2**, and DMPC-**3** vesicles, respectively. Zeta potentials were measured at pH = 6.86 at 25 °C.

### 3.6. Fluorescence autocorrelation curve measurements

Fluorescence autocorrelation curves were measured on a compact FCS apparatus (C9413-01MOD, Hamamatsu Photonics K. K., Japan) with 8-well LabTek chambers (Nalge Nunc International, Rochester, NY) at room temperature. The excitation power at the objective lens was 30 mW. Measurement time and sample volume for FCS were 30 s and ca. 0.5 mL, respectively. The confocal pinhole diameters were adjusted to 50 μm. The emission signals were detected at > 568 nm.

## 4. Conclusions

So far, the binding properties of *p*-sulfonatocalix[n]arenes and its related compounds have been studied in homogenous aqueous solution. We synthesized amphiphilic derivatives of *p*-sulfonatocalix[n]arenes and studied their binding ability and selectivity in a lipid bilayer vesicle system. The measurements of fluorescence spectra and fluorescence anisotropy of Rh6G based on competitive fluorophore displacement experiments suggested that ACh binds to the surface of DMPC-**3** vesicles. However, the experimental data obtained from the steady state fluorescence technique could not afford the direct evidence for the ACh binding to the vesicles. By using FCS, we could show that DMPC vesicles containing **3** have interfacial recognition ability for ACh. In this respect, FCS was a very useful method to clarify whether ACh can bind to DMPC vesicles containing **1-3**. From the analysis of fluorescence autocorrelation curves of Rh6G, the binding selectivity of the DMPC-**3** vesicle was determined: ACh > choline ∼l-agrinine > l-lysine ∼ NH_4_^+^ > l-histidine ≫ l-aspartic acid∼ l-glutamine∼GABA The binding selectivity of the DMPC-**3** vesicle was not directly correlate to that of the parent *p*-sulfonatocalix[8]arene in homogenous solution. This result suggests that in a vesicle system, the membrane potential would play an important role in the binding selectivity of receptors incorporated into the vesicle. To the best of our knowledge, amphiphilic *p*-sulfonatocalix[8]arene **3** is the first artificial ACh receptor that works in a lipid bilayer membrane system.

## Figures and Tables

**Table 1.** Zeta potentials of vesicles in 10 mM phosphate buffer (pH = 6.86).

**Zeta potentials (mV)**			
DMPC	DMPC-**1**	DMPC-**2**	DMPC-**3**
1.6 ± 0.6	-10.4±2.3	-8.2±1.0	-7.1±0.8
